# Induction of Redox-Mediated Cell Death in ER-Positive and ER-Negative Breast Cancer Cells by a Copper(II)-Phenolate Complex: An In Vitro and In Silico Study

**DOI:** 10.3390/molecules25194504

**Published:** 2020-10-01

**Authors:** Vaiyapuri Subbarayan Periasamy, Anvarbatcha Riyasdeen, Venugopal Rajendiran, Mallayan Palaniandavar, Hanumanthappa Krishnamurthy, Ali Abdullah Alshatwi, Mohammad Abdulkader Akbarsha

**Affiliations:** 1 Department of Animal Science, Bharathidasan University, Tiruchirappalli 620024, India; vsperrys@gmail.com (V.S.P.); anfariyas@gmail.com (A.R.); 2 Department of Food and Nutrition, College of Food and Agricultural Sciences, King Saud University, Riyadh 11451, P.O. Box 2460, Saudi Arabia; alshatwi@ksu.edu.sa; 3 Department of Chemistry, School of Basic and Applied Sciences, Central University of Tamil Nadu, Thiruvarur 610005, India; rajendiran@cutn.ac.in; 4 Department of Chemistry, Bharathidasan University, Tiruchirappalli 620024, India; palaniandavarm@gmail.com; 5 National Centre for Biological Sciences, Tata Institute for Fundamental Research, Bangalore 560065, India; krishna@ncbs.res.in; 6 Mahatma Gandhi-Doerenkamp Center (MGDC) for Alternatives to Use of Animals in Life Science Education, Bharathidasan University, Tiruchirappalli 620024, India; 7 Research Co-ordinator, National College (Autonomous), Tiruchirappalli, 620001, India

**Keywords:** anticancer agents, apoptosis, breast cancer, cell cycle arrest, copper(II)-phenolate complex, necrosis, ROS

## Abstract

This research was aimed at finding the cytotoxic potential of the mixed ligand copper(II) complex [Cu(tdp)(phen)](ClO_4_)—where H(tdp) is the tetradentate ligand 2-[(2-(2-hydroxyethylamino)-ethylimino)methyl]phenol, and phen is 1,10-phenanthroline—to two genotypically different breast cancer cells, MCF-7 (p53^+^ and ER^+^) and MDA-MB-231 (p53^-^ and ER^-^). The complex has been already shown to be cytotoxic to ME180 cervical carcinoma cells. The special focus in this study was the induction of cell death by apoptosis and necrosis, and its link with ROS. The treatment brought about nuclear fragmentation, phosphatidylserine externalization, disruption of mitochondrial trans-membrane potential, DNA damage, cell cycle arrest at sub-G1 phase, and increase of ROS generation, followed by apoptotic death of cells during early hours and a late onset of necrosis in the cells surviving the apoptosis. The efficacy of the complex against genotypically different breast cancer cells is attributed to a strong association through p53-mitochondrial redox—cell cycle junction. The ADMET properties and docking of the complex at the active site of Top1 are desirable attributes of a lead molecule for development into a therapeutic. Thus, it is shown that the copper(II)–phenolate complex[Cu(tdp)(phen)]^+^ offers potential to be developed into a therapeutic for breast cancers in general and ER-negative ones in particular.

## 1. Introduction

Therapeutic potential of metal-based complexes has been of great interest in the drug discovery scenario due to their unique electronic, thermodynamic and kinetic properties such as redox activity, metal–ligand exchange interactions, high electron affinity, and 3D geometry, which favor targeting specific biomolecules [1]. These unique pharmacodynamic characters of metal-based complexes have become an attractive therapeutic concept for the past few decades towards development of novel targeted drug molecules [2]. Currently, platinum-based analogues are among the most practiced main-line metal-based drugs for treatment of a variety of cancers including breast cancer [3,4]. However, these drugs are not very selective to cancers since normal cells are also affected, which leads to substantial dose-limiting acute and chronic toxicities [5,6]. It is generally believed that non-platinum drugs have the potential to overcome this shortcoming [7,8].

Complexes of metals such as platinum, gold, palladium, ruthenium, rhodium, copper, and lanthanum, with aromatic N-containing ligands (pyridine, pyrazinamide, nicotinamide, imidazole, nicotinic acid, 1,10-phenanthroline, and their derivatives), have been shown, using in vitro and in vivo models, to offer promise as anticancer, antibacterial, antiviral, and antifungal agents [9,10,11,12,13,14,15,16,17,18,19]. Among these non-Pt compounds, copper complexes are particularly attractive for designing lead anticancer agents, owing to unique pharmacodynamic properties, enhanced bioavailability, and less toxicity [20,21,22]. Copper is an essential, endogenous metal and is found in the many proteins and metalloenzymes that are involved in enhancement of bioavailability and driving cellular redox reactions that target specific pathways without producing side effects [23,24].

Aiming at these desired pharmacodynamic profiles, diimines, tetradentate ligands and substituted derivatives as ligands are currently attractive in the discovery of metal-based drugs for different cancers [25,26]. These coordinated or transition metal complexes have been found to be potent as cell cycle inhibitors [27,28,29], DNA topoisomerase inhibitors [30,31], pro- and anti-apoptotic protein modulators (p53, Bax and Bcl-2) [32,33,34], etc. Particularly, these metal complexes bring about cancer cell death through inhibition of DNA synthesis [14,26,28,35], alteration of mitochondrial membrane potential and/or suppression of inhibitors of apoptosis [26,36,37]. In addition, diimine ligands of transition metals such as copper are advantageously tuned by increased transport of the metal complexes into the cells [38,39].

In this study, we used copper(II) coordinated by 1,10-phenanthroline (phen) chelator, which can exhibit effective and targeted activity due to the ability of the diimine ligand to engage in DNA recognition. Unique 3D geometry and electronic and other molecular mechanics of this specially designed complex can selectively regulate the membrane permeability, bind with DNA and induce cell death (i.e., apoptosis) [9]. Also, the typical structure of diimines provides for the ability of the complex to participate as a DNA intercalator and/or nucleo-cytoplasmic disruptor through apoptotic mechanism [28,29].

The currently available main line cancer chemotherapeutics are endowed with potential to induce apoptosis in various cancers. Programmed cell death (including apoptosis), as a distinct series of cellular pathways, offers unique targets for chemotherapeutic intervention [40,41,42,43,44]. The direct molecular link between cancer pathogenesis and dysregulation of programmed cell death (PCD) can be used as a therapeutic target by triggering PCD in cancers with drugs such as copper complexes. Especially, cell death mechanisms such as apoptosis, autophagy and necro-apoptosis are different kinds of programmed cell death (PCD) and brilliant cell physiological mechanisms by which cells with irreparably damaged DNA are removed without any inflammatory response [40,43].

Most of the studies emphasize that copper phenolate complexes can act as programmed cell death (apoptosis) modulators via triggering different signal transduction pathways (such as p53 and/or caspase-dependent apoptosis), which provide various hypothetical opportunities to control and/or select pathways in order to find the mode of action [34,37,45,46].

Especially, estrogen receptor (ER) status is one of the principal foci for treatment of breast cancer. The presence or absence of ER in the breast cancer cell is a valuable prognostic factor that provides predictive value to the potential benefits of target-based therapies [47]. Approximately 70% of metastatic breast cancers are ER-positive, and the patients tend to have a greater chance of effective tumor response and longer survival than patients with ER-negative cancers [48,49,50].

Some time back, our group synthesized a number of copper(II)–phenolate complexes and evaluated the in vitro anti-proliferative activity with respect to the cervical carcinoma cell ME180 [51]. Since this cell contains HPV18 DNA [24], we picked up interest in finding the efficacy of these copper(II)–phenolate complexes in cancer cells that are not transformed by viral DNA and, hence, chose to conduct the study in breast cancer cells that are genotypically different among themselves.

In the present study, we elucidated the cell death-inducing property of one of the copper(II)–phenolate complexes, [Cu(tdp)(phen)]^+^, in MCF-7 (ER-positive and p53-positive) and MDA-MB-231 (ER-negative and p53-negative) cells, adopting different methods with special reference to screening of apoptosis. This copper complex brought about extensive changes in both the cell types, indicating apoptosis during the early hours of treatment and necrosis during the late hours, with the molecular mechanisms of action apparently selective for each of the two cell types. In silico prediction of ADMET and molecular docking indicate desirable features.

## 2. Materials and Methods

### 2.1. Copper Complexes

**Figure 1:**
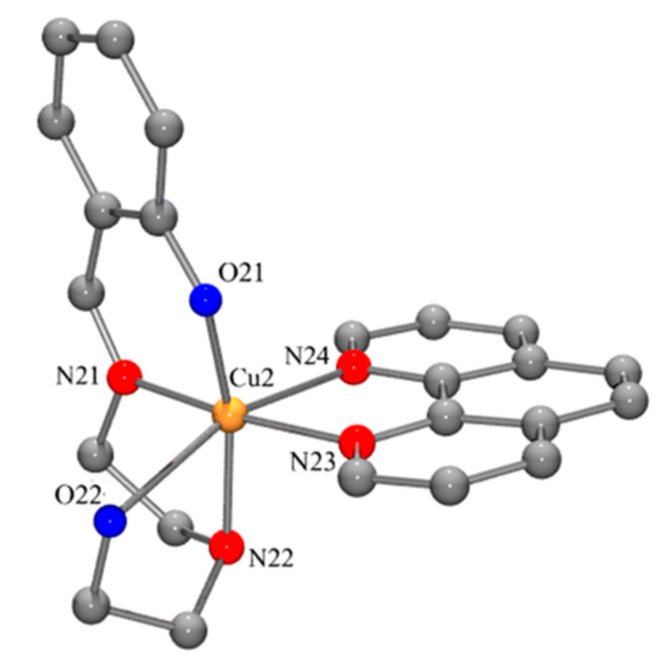
Ball-and-stick representation of the crystal structure of [Cu(tdp)(phen)](ClO_4_)·CH_3_OH in which atoms are shown as spheres of arbitrary diameter. Hydrogen atoms are omitted for purpose of clarity [51].

The H(tdp) and [Cu(tdp)(phen)]ClO_4_·CH_3_OH were synthesized and characterized according to the procedures and methods described in our previous paper [51]. For comprehending the structure–activity relationship, the crystal structure of the complex is shown in Figure 1.

### 2.2. Cell Culture

Breast cancer cells MCF-7 (ER-positive and p53-positive) and MDA-MB-231 (ER-negative and p53-negative) were obtained from National Centre for Cell Science (NCCS), Pune, India. The cells were cultured in DMEM (Sigma-Aldrich, St. Louis, MO, USA) supplemented with 10% fetal bovine serum (Sigma-Aldrich) and 100 U/mL penicillin and 100 μg/mL streptomycin as antibiotics (Himedia, Mumbai, India), in T25 or T75 flasks and 6, 12, 24, or 96-well cell culture plates (TPP, Trasadingen, Schaffhausen, Switzerland), depending on the context, at 37 °C, in a humidified atmosphere of 5% CO_2_ in a CO_2_ incubator (Heraeus, Hanau, Germany). All experiments were conducted using cells from passage 15 or less.

### 2.3. Cytotoxicity Assay

MTT assay was performed as described in the previous study [52]. The copper complex was prepared as stock solutions, to suit the universal 96-well plate map, at different concentrations in nM or µM, dissolved in 100% DMSO (Sigma-Aldrich, St. Louis, MO, USA) or dH_2_O. Working solutions were prepared in the culture medium, at a final DMSO concentration of 0.02%, where DMSO was used as the solvent, and added to the wells 24 h after seeding of 5 to 7 × 10^4^ cells per well in 200 μL of fresh culture medium. DMSO (0.02%) was used as the solvent control. Light microscopic cytological changes were monitored and photographed following exposure to different concentrations of the complex for 24 and 48 h using an inverted microscope (Carl Zeiss, Jena, Germany). After the respective periods of treatment, 20 μL of MTT solution [5 mg/mL in phosphate-buffered saline (PBS)] was added to each well, and the plates were wrapped with aluminum foil and incubated for overnight at 37 °C. The purple formazan crystal that formed was dissolved in 100 μL of 100% DMSO. The absorbance of the color was monitored at 570 nm (measurement) and 630 nm (reference) using a 96-well microplate reader (Bio-Rad, Hercules, CA, USA). Data were collected for four replicates each and used to calculate the median effect dose, i.e., IC_50_ (Dm value), sigmoidity of the dose-effect curve (m value) and linear correlation coefficient of the median-effect plot (r value), using Calcusyn software (Biosoft, Cambridge, UK).

### 2.4. AO & EB Fluorescent Probe for Assessment of Cell Death

Acridine orange (AO) and ethidium bromide (EB) dual staining was performed as described by Spector et al. [53]. The cells treated with the complex at the respective IC_50_ concentrations for 24 and 48 h were incubated with AO and EB solution (1 part each of 100 μg/mL AO and EB in PBS) and mixed gently, and examined in a fluorescent microscope (Carl Zeiss, Jena, Germany) using a UV filter (450–490 nm). Three hundred cells per sample were counted in duplicate for each dose point. Cells were scored as viable, apoptotic or necrotic, as judged from nuclear morphology and membrane integrity, and the respective percentages of apoptotic and necrotic cells were then calculated. The cells of interest were photographed.

### 2.5. Annexin V-Cy3 Apoptosis Assay

Phosphatidylserine translocation from inner to outer leaflet of the plasma membrane is one of the early features of apoptosis. Cell surface phosphatidylserine was detected by phosphatidylserine-binding protein annexin V conjugated with Cy3 using the commercially available annexin V-Cy3 apoptosis detection kit (APOAC, Sigma-Aldrich, St. Louis, MO, USA ). Cells treated with 24 h IC_50_ of the complex for 6, 12 and 24 h were washed with cold phosphate-buffered saline and incubated with 50 µL of the double label-staining solution (containing 1 µg/mL annexin-Cy3 and 100 µM 6-carboxyfluorescein diacetate) for 10 min at room temperature in the dark. The cells were then washed with 1× binding buffer, and 300 cells at random were observed in the fluorescent microscope. The combination of 6-carboxyfluorescein diacetate (6-CFDA) with annexin V conjugated with Cy3 facilitated detection of live cells (green), necrotic cells (red), and apoptotic cells (red nuclei and green cytoplasm). The percentage of cells reflecting cell death (apoptotic and necrotic, separately) was calculated. Data were collected from two individual experiments, each in duplicate, and used to calculate the respective means and the standard deviations.

### 2.6. Measurement of ROS

Intracellular reactive oxygen species (ROS) was determined by using the fluorescent probe 2′,7′-dichlorofluorescein diacetate (DCFH-DA) [54]. DCFH-DA readily diffuses through the cell membrane and is enzymatically hydrolyzed by intracellular esterases to form non-fluorescent 2′,7′-dichlorofluorescein (DCFH), which is then rapidly oxidized to form highly fluorescent 2′,7′-dichlorofluorescin (DCF) in the presence of ROS. The DCF fluorescence intensity is indicative of the amount of ROS formed intracellularly. Here, after treating the cells with IC_50_ of the complex for 6, 12 and 24 h, the medium was poured out, the cells were washed with PBS and then incubated with 10 µM DCFH-DA in the loading medium. After DCFH-DA was removed, the cells were washed with PBS and lysed by sonication for 1 min at room temperature. Immediately, the fluorescence intensity (relative fluorescence units) was measured at 485 nm excitation and 530 nm emission in a Jasco (Easton, MD, USA) F 6500 spectrofluorometer.

### 2.7. Assay of Mitochondrial Trans-Membrane Potential (JC-1 Staining)

Mitochondrial trans-membrane potential was measured using the fluorescent probe JC-1, which produces green fluorescence in the cytoplasm and red to orange fluorescence when concentrated in mitochondria that are metabolically active and so maintain a negative internal potential [53]. JC-1 staining was performed according to the manufacturers’ protocol (Sigma, St. Luis, MI, USA). Cells treated for 6 and 12 h with 24 h IC_50_ of the complex in 12-well plates were incubated for 30 min with JC-1 (2 µg/mL) prepared in the culture medium. The adherent cell layer was then washed with PBS and examined in the fluorescent microscope using a UV filter (450–490 nM). Three hundred cells per sample were counted, in duplicate, for each dose- and time point. Orange-red fluorescence of cells indicated intact mitochondrial membrane whereas green fluorescence indicated loss of mitochondrial membrane integrity. These specific fluorescent patterns were indicative of live and dead cells, respectively (if dead, apoptotic or necrotic).

### 2.8. Alkaline Single-Cell Gel Electrophoresis (Comet) Assay

DNA damage was quantified adopting comet assay [55]. Cells treated with the complex, at the 24 h IC_50_ concentration, for 12 and 24 h, were trypsinized and brought to suspension. Two hundred micro-liters of 1% normal agarose in PBS at 65 °C was dropped gently on to a fully frosted microslide, covered immediately with a cover slip and placed over frozen ice pack for about 5 min. The cover slip was removed after the gel had set. The cell suspension was mixed with 1% low melting agarose at 37 °C in 1:3 (*v/v*) ratio. One-hundred micro-liters of this mixture was applied quickly on top of the gel, coated over the micro-slide and allowed to set as before. A third coating of 100 μL 1% low melting agarose was laid on the gel containing the cell suspension and allowed to set. The slides were prepared in tetraplicate for each cell fraction. After solidification of agarose, the cover slips were removed and the slides were immersed in ice-cold lysis solution (2.5 M NaCl, 100 mM Na_2_EDTA, 10 mM Tris, 0.1% Triton X-100; pH adjusted to 10 using NaOH pellets) and placed in a refrigerator at 4 °C for 16 h. The slides, after being removed from the lysis solution, were placed horizontally in an electrophoresis tank. The reservoirs were filled with electrophoresis buffer (300 mM NaOH, 1 mM Na_2_EDTA, pH > 13) until the slides were just immersed in it. The slides were allowed to stand in the buffer for about 20 min (to allow DNA unwinding) after which electrophoresis was carried out at 0.8 v/cm for 15 min. After electrophoresis, the slides were removed, washed thrice in neutralization buffer (0.4 M Tris, pH 7.5) and gently dabbed to dry. The nuclear DNA was stained with 20 µL EB (50 µg/mL). Photographs were obtained in the fluorescent microscope (450–490 nm). One-hundred-and-fifty cells from each treatment were digitalized and analyzed using CASP comet assay software. The images were used to determine the DNA content of individual nuclei and evaluate the degree of DNA damage representing the fraction of total DNA in the tail. Cells were assigned to five classes, intact (0–20%), slightly damaged (20–40%), damaged (40–60%), highly damaged (60–80%), and dead (80–100%).

### 2.9. Cell Cycle Analysis

Flow cytometer-based DNA content analysis is an important tool to detect cell death/cell cycle arrest. Cell cycle analysis was performed as per an established protocol [56]. The cells were seeded in T_25_ flasks at a density of 1 × 10^6^ cells/flask. After 24 h, the complex, at the respective IC_50_ concentrations, was added to the flasks and incubated for 24 and 48 h. The cells were trypsinized, harvested, fixed in 80% cold ethanol, and stored for 24 hr at 4 to 8 °C. The cells were rehydrated with PBS and centrifuged at 4000 rpm for 5 min. Further, the cell pellets were resuspended in propidium iodide (50 µg/mL). Cell cycle distribution was analyzed using FACScan (Becton-Dickinson, Sanjose, CA, USA) with 15 mw, 488 nm argon ion laser. Propidium iodide signals were collected using 585/42 band pass filter. The data were analyzed using Cell Quest software.

### 2.10. Western Blot Analysis

Total proteins from untreated and treated cells were extracted, quantified and subjected to electrophoresis, followed by Western blot analysis [57,58] using antibodies of specific apoptotic markers *viz*., p53, Bcl-2 and Bax, with β-actin as the loading control (Sigma-Aldrich, St. Louis, MO, USA). In T_75_ flasks, 5 × 10^6^ cells were seeded and treated with the complex at the 24 h IC_50_ concentration for 6, 12, and 24 h. The cells were washed in cold PBS and lysed in RIPA buffer with 1% Triton X-100, 50 mM Tris, pH 7.6, and 150 mM NaCl containing 2 mM phenylmethylsulfonyl fluoride. Total protein of the lysate was quantified adopting Bradford assay [57]. Subsequently, the proteins were separated adopting sodium dodecyl sulfate-polyacrylamide gel electrophoresis and electroblotted to a nitro-cellulose membrane. The loading and transfer of equal amounts of protein were confirmed by staining the membrane with PonceauS. The membranes were blocked for 3 h using 5% non-fat dry milk powder in Tris-buffered saline (TBS: 10 mM Tris-HCl pH 7.4, 100 mM NaCl) and then immunoblotted for 3 h with the primary antibody at appropriate dilutions. The membranes were washed with TBS/0.05% Tween-20 and incubated with secondary antibodies conjugated with peroxidase-conjugate (Bangalore Genei, Bangalore, India) and/or alkaline phosphatase for 1 h. After extensive washing, the reaction product was developed using diaminobenzidine (DAB) substrate. The Western blot image was digitized, normalized, and subjected to image analysis using ImageJ (NIH, Bethesda, MD, USA) software (Open Access Public Domain).

### 2.11. ADMET and Molecular Docking Studies

Different ADMET and molecular properties such as physicochemical parameters, pharmacokinetics, lipophilicity, water solubility, carcinogenicity, mutagenicity, and relevant descriptors of the copper complex were predicted using Discovery Studio 3.5. For the molecular docking study, 2D structure of the copper complex was drawn using ChemDraw tools. Further ligand preparation, including 3D structure conversion and minimization, was carried out as per the Discovery Studio v3.5 protocol. The 3D structures of topoisomerase I (Top1) with DNA complex (PDB id: 1SC7) were retrieved from the RCSB Protein Data Bank. Target protein preparation and minimization were carried out as described in the Discovery Studio protocol. The optimal target position and different interactive bonds of the copper complex with Top1 were determined using the Molegro Virtual Docker software (trial version). Further, bonding interactions between the copper complex and Top1 were visualized by Discovery Studio Visualizer.

### 2.12. Statistical Analysis

The results were analyzed for IC_50_ values, sigmoidity, correlation and mean ± standard deviation using Calcusyn software and Microsoft Excel 2000 software. For analysis of paired samples, Student’s *t*-test was conducted, and *p* value <0.05 was considered statistically significant. The ROS data were subjected to Mann Whitney non-parametric test at 90% confidence limit using Graphpad Prism software.

## 3. Results

### 3.1. Cytotoxic Potential of the Complex as Revealed in MTT Assay

The cytotoxic potential of the complex on human breast cancer cell lines was determined as the dose value of exposure of the complex required to reduce survival of the cells to 50% (IC_50_). The copper complex produced time- and concentration-dependent cytotoxic effects in both breast cancer cells. The IC_50_ values, sigmoidity and correlation are presented in Table 1. The IC_50_ value for the complex was low for both the cell lines at 48 h treatment compared to 24 h treatment. Further, at 48 h time point it was relatively low for MDA-MB-231 cells (1.0 ± 0.9 µM), compared to MCF-7 cells (1.2 ± 0.8 µM), though the difference was not statistically significant.

### 3.2. Indications of Apoptosis and Necrosis as Revealed in AO-EB Staining

**Figure 2:**
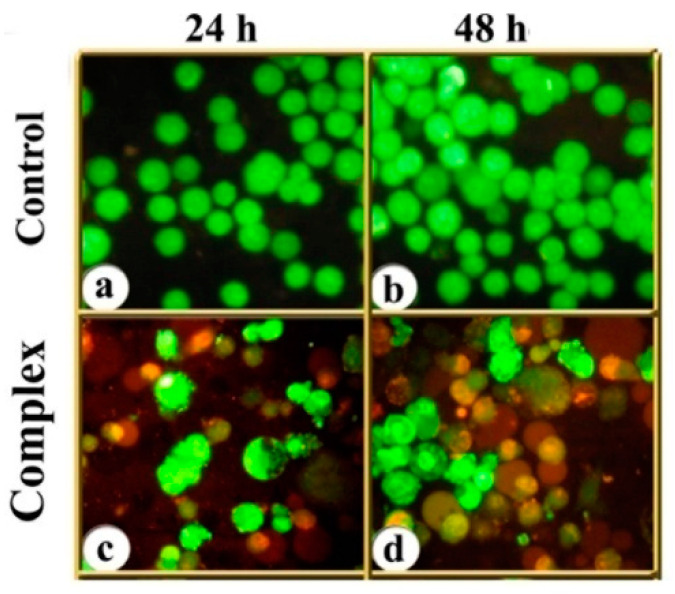
MCF-7 cells treated with the complex for 24 and 48 h and AO & EB stained. (**a**,**b**) control cells (viable; uniform green-fluorescence); (**c**,**d**) cells treated with the complex; pre-apoptotic (yellow-fluorescence) and apoptotic (bright orange fluorescence) cells. Necrotic cells are swollen and fluoresce bright-red (400×).

After the cells were exposed to the respective IC_50_ concentrations of the complex for 24 and 48 h, and stained with AO & EB, the cells were assigned to four patterns according to the fluorescence emission and nature of chromatin condensation in the nuclei (Figure 2): (i) viable (uniformly green-fluorescing nuclei with a highly organized structure), (ii) early apoptotic (still had intact membranes but DNA fragmentation was initiated and had green-fluorescing nuclei, and peri-nuclear chromatin condensation was visible as bright green patches or fragments), (iii) late apoptotic (with bright orange fluorescing nuclei with condensed or fragmented chromatin), and (iv) necrotic cells (uniformly bright red-fluorescing nuclei with no indication of chromatin fragmentation but the cells were swollen to a large size).

**Figure 3:**
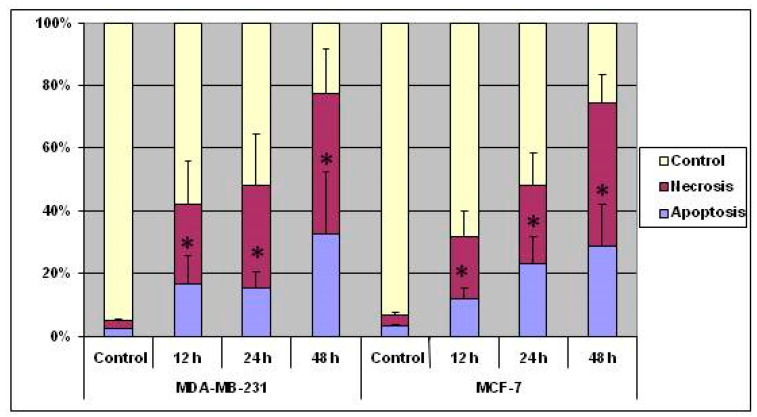
Data show the response of MCF-7 and MDA-MB-231 cells, in terms of apoptosis (AO & EB staining), to treatment with the copper complex. The percentages of cells in apoptosis and necrosis are indicated by the histograms. The data shown are the means from triplicates. Vertical bars represent standard error of the mean. *p*-values obtained between control and complex-treated cells using *t*-test where ‘*’ indicates (*p* ≤ 0.05)—significant.

Over all, the results indicated that treatment with the copper complex induced both MCF-7 and MDA-MB-231 cells to take to death through apoptosis as well as necrosis (Figure 3), and the response was dependent on the cell type, concentration of the complex, and duration of the treatment.

### 3.3. Indications of Early Apoptotic Changes as Revealed in Annexin V-Cy3 and 6-CFDA Staining

**Figure 4:**
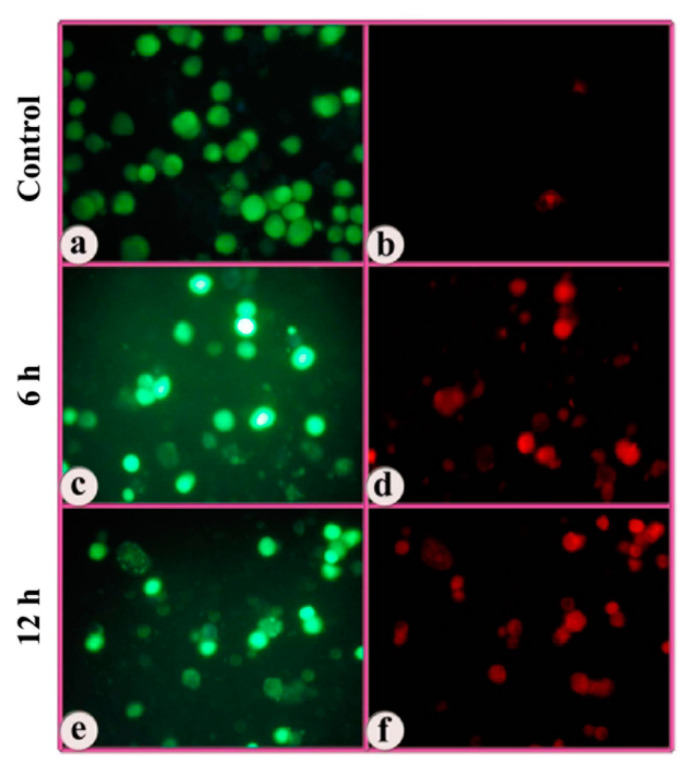
Fluorescent photomicrographs showing annexin V-Cy3 apoptosis assay on MCF-7 breast cancer cells treated with the complex. (**a**,**b**) control (untreated); (**c**,**d**) treated with the complex for 6 h; (**e**,**f**) treated with the complex for 12 h.

**Figure 5:**
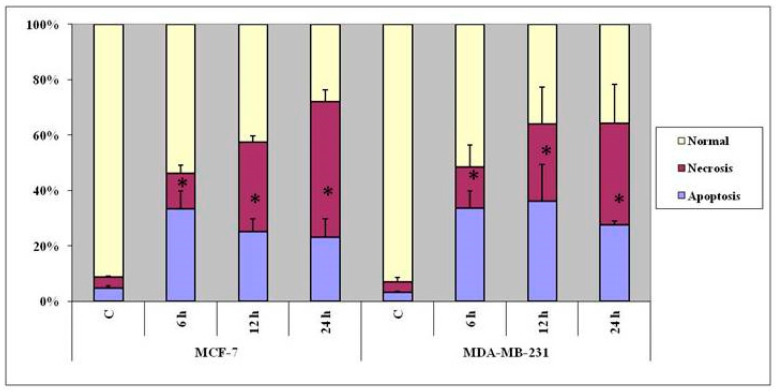
Data show the response of MCF-7 and MDA-MB-231 cancer cells, in terms of apoptosis (annexin V-Cy3 staining), to treatment with the complex. The percentages of cells in apoptosis and necrosis are indicated by the histograms. The data shown are means from triplicates. Vertical bars represent standard error of the mean. *p*-values obtained between control and treated cells with the complex using *t*-test where ‘*’ indicates (*p* ≤ 0.05)—significant.

The cells treated with the complex were subjected to dual staining with annexin V-Cy3 and 6-CFDA to obtain evidence for early apoptotic changes. Annexin V binds to phosphatidylserine moieties that become exposed on the outer surface of the cell membrane during apoptosis, whereas 6-CFDA staining serves as a marker for viable cells. This combination helps to differentiate early apoptotic cells (annexin V-positive, 6-CFDA-positive), necrotic cells (annexin V-positive, 6-CFDA-negative), and viable cells (annexin V-negative, 6-CFDA-positive). Both MCF-7 and MDA-MB-231 cells treated with the complex exhibited significant incidence of apoptosis (Figure 4 and Figure 5).

Here again, it appeared that the mode of cell death was dependent on incubation time from two perspectives, (i) with an increase in incubation time, more cells died; and (ii) with lesser incubation time, the incidence of apoptosis was more than necrosis, and with longer incubation time, more cells took to necrosis than apoptosis. Within this generalization, the incidence of apoptosis was more in p53^−^& ER^−^ MDA-MB-231 cells than p53^+^ & ER^+^ MCF-7 cells, but this difference was not statistically significant.

### 3.4. Changes in ROS Level

**Figure 6:**
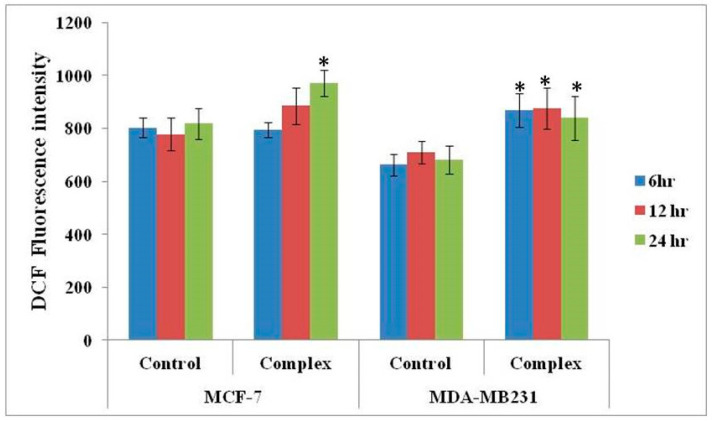
Cellular ROS levels in MCF-7 and MDA-MB-231 cancer cells treated with the complex at 24 h IC_50_ for 6, 12 and 24 h. *p*-values obtained between control and treated cells with the complex using Mann Whitney non-parametric test where ‘*’ indicates (*p* ≤ 0.05)—significant.

MCF-7 and MDA-MB-231 cells treated with the complex at 24 h IC_50_ concentration for 6, 12 and 24 h were subjected to analysis of cellular ROS levels. The treatment induced both the cell types to generate high amounts of ROS, in a manner duration-dependent with regard to MCF-7 cells. However, data were tested at 90% confidence level. For MCF-7 cell significance was revealed only for the 24 h time point, whereas for MDA-MB-231, data is significant at all three time points (Figure 6).

### 3.5. Change in the Mitochondrial Membrane Potential as Revealed in JC-1 Staining

**Figure 7:**
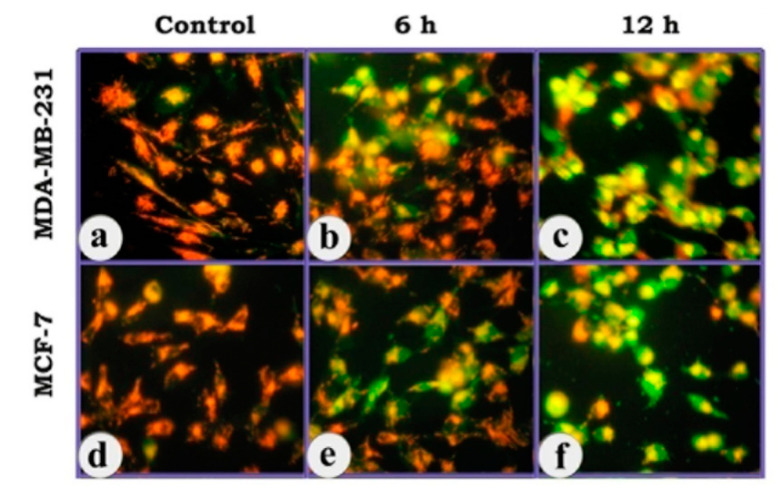
Photomicrographs of breast cancer cells (MCF-7) showing the mitochondrial trans-membrane potential as revealed in JC-1 staining. (**a**,**d**) control; JC-1 dye accumulation in the mitochondria of healthy cells as aggregates (red- to orange-fluorescence); (**b**,**e**) cells treated with the complex for 6 h; (**c**,**f**) cells treated with the complex for 12 h. (400×). Due to collapse of mitochondrial transmembrane potential, the JC-1 dye remains in the cytoplasm in the monomeric form and emits green fluorescence (6 & 12 h).

The fluorescent cationic dye JC-1 was used to detect the change in mitochondrial trans-membrane potential, which is an early event in the induction of apoptosis. In healthy, non-apoptotic cells the dye accumulates and aggregates within the mitochondria, resulting in bright orange to red fluorescence. In apoptotic cells, due to collapse of mitochondrial trans-membrane potential, JC-1 remains outside the mitochondria, in the cytosol, in the green-fluorescent monomeric form. The results revealed that there was dramatic change in mitochondrial trans-membrane potential in both MCF-7 and MDA-MB-231 cells on treatment with the copper complex, as revealed in the intense green fluorescence of the cytosol in a duration-dependent manner, indicating the onset of an early event in the apoptosis cascade (Figure 7).

### 3.6. DNA Damage as Revealed in Comet Assay

**Figure 8:**
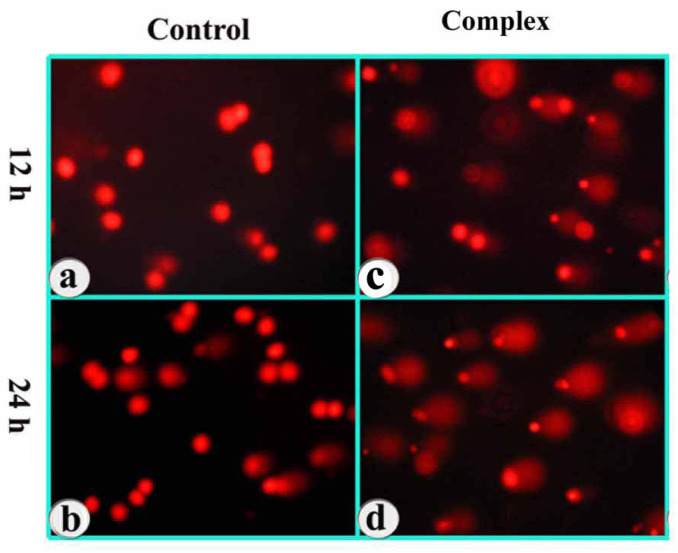
Images of DNA double-strand breaks (comets) at 12 and 24 h treatment of MCF-7 cells with the complex. Left panel (**a**,**b**)—control; right panel (**c**,**d**)—treated. The duration-dependence of the DNA damage is revealed.

The DNA content of individual nuclei and the degree of DNA damage representing the fraction of total DNA in the tail were determined adopting comet assay, and the cells were assigned to five classes, referred to as *vide supra.* A significant percentage of cells showed increased length and density of comet tails in a time-dependent manner as compared to untreated cells (Figure 8).

**Figure 9:**
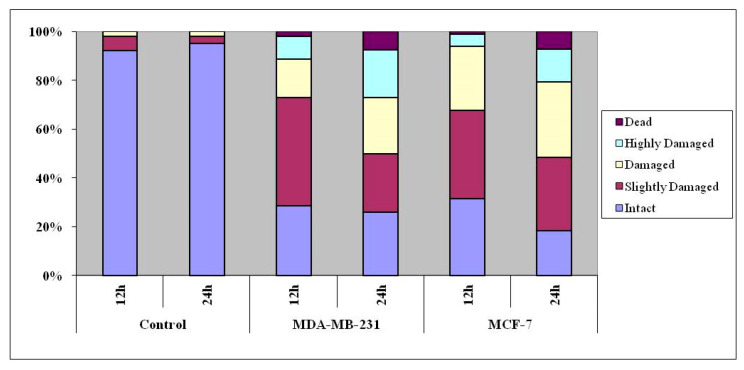
Results of comet assay in MCF-7 and MDA-MB-231 cancer cells treated with the complex for 12 and 24 h. The 100% stacked column compares the percentage of comet DNA.

As seen in Figure 9, a fairly high percentage of cells treated with the complex got assigned to ‘damaged’, ‘highly damaged’ and ‘dead’ categories at 12 and 24 h treatment, the response increasing in a duration-dependent manner. Here, the ‘dead’ and ‘highly damaged’ cell populations were more among ER-negative MDA-MB-231 cells than ER-positive MCF-7 cells.

### 3.7. Change in Cell Cycle Progression as Revealed in Flow Cytometric Analysis

**Figure 10:**
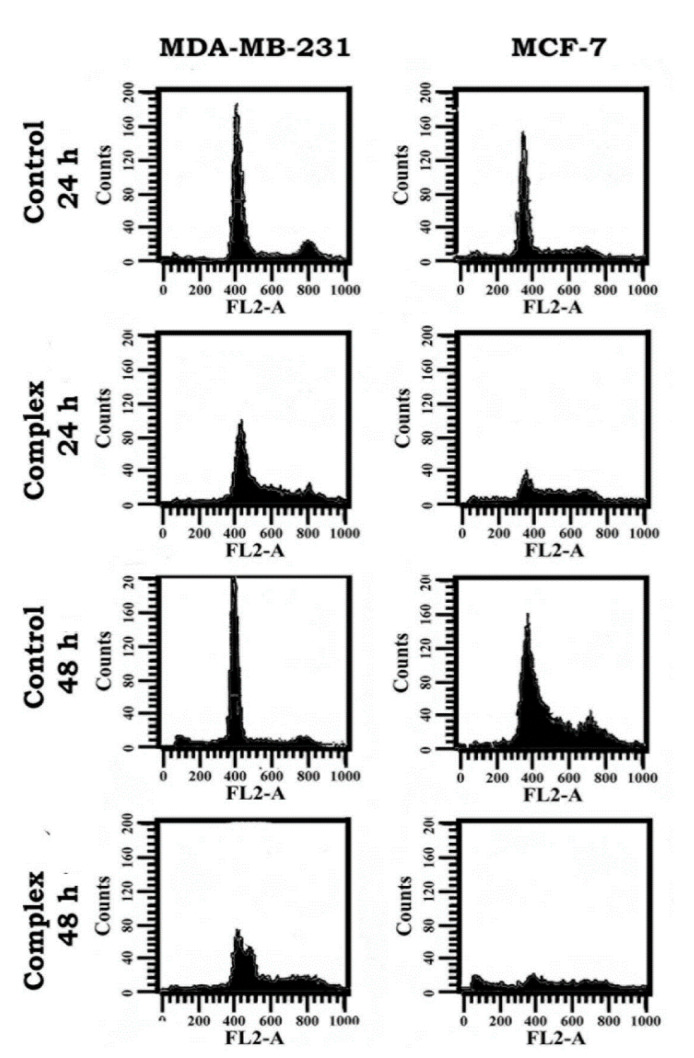
Histograms derived from flow cytometry data showing the effect of the complex on cell cycle distribution of breast cancer cells (MDA-MB-231 & MCF-7) for 24 and 48 h treatment.

Since the cells treated with the complex indicated a fairly good response in terms of apoptosis, the complex was analyzed for impact on the cell cycle progression, adopting flow cytometry. The DNA of 10,000 cells of each sample was evaluated, and the results revealed duration-dependent distribution of both the breast cancer cells belonging to the treatment groups different from the respective controls (Figure 10). As seen in Table 2, there was significant increase of cells in the S- and G_2_/M phases when treated with the complex, showing that there was significant arrest of cell division at these respective phases. Importantly, this response was more dramatic in MDA-MB-231 cells than MCF-7 cells.

### 3.8. Change in Molecular Indicators of Apoptosis

**Figure 11:**
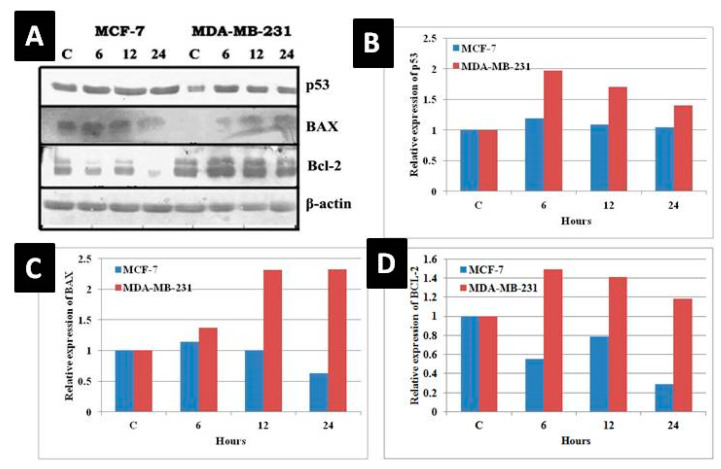
Western blot analysis of modulation of pro- and anti-apoptotic proteins due to treatment of MCF-7 and MDA-MB-231 cells with the complex. (**A**) The blot was probed with antibodies for p53, Bcl-2, and Bax, where β-actin was the loading control. (**B**–**D**) The densitometry graphs represent the relative expression of p53, BAX and Bcl-2 proteins, respectively.

The expression of p53 (tumor suppressor protein) and two Bcl-2 family members, Bcl-2 (anti-apoptotic) and Bax (pro-apoptotic), was evaluated adopting Western blot technique (Figure 11). Using p53 wild-type MCF-7 and p53-mutant MDA-MB-231 cells, a strong correlation was found between p53 status and the response of the cells to the complex in terms of apoptosis. In the p53-positive MCF-7 cells, the treatment produced a slight increase in the p53 protein expression at 6 and 12 h time points, which harmonized with Bax up-regulation and Bcl-2 down-regulation. In the p53-mutant MDA-MB-231 cells, p53 protein expression was increased at all three time points with a corresponding increase of Bax protein, but Bcl-2 protein expression also remained fairly high.

### 3.9. ADMET and Molecular Docking Studies

In silico-based molecular and ADMET properties such as distribution parameters (AlogP98); solubility; absorption level; absorption parameter (PSA-2D); plasma protein binding (PPB); blood brain barrier (BBB); cytochrome P450 2D6 (CYP2D6); hepatotoxicity; Ames_Prediction; Ames_Score; TOPKAT FDA Carcinogenicity, Female Rat; and TOPKAT FDA Carcinogenicity, Male Rat, compared and interpreted based on the references, were predicted for our copper complex using Drug Discovery Studio 3.5 (Table 3). The values of molecular properties, such as AlogP and solubility, can help us evaluate and predict the hydrophilicity or lipophilicity of a compound. The predicted partition coefficient AlogP98 (3.588) and aqueous solubility (score = −7.391) of our copper complex assign to the hydrophobic scale, which can be favorable for membrane permeability. The predicted parameter scores of absorption level (scale = 0) and PSA-2D (31.909) indicate good absorption in the human intestine. The plasma protein binding (PPB) (score = 2.89411) suggests that our copper complex likely binds to carrier proteins in the blood. The predicted BBB value (score = 0.45) is a clear indication of penetrability through the blood brain barrier. The prediction of the complex based on Ames (v3.1) score (−2.33954), hepatotoxicity (−2.52683), and CYP2D6 enzyme inhibition score (−2.30385) shows it to be in the non-toxic range. TOPKAT carcinogenicity in female rat (v3.1) and TOPKAT carcinogenicity in male rat model (v3.1) predictions indicate the complex to be non-carcinogenic. Overall, these pharmacokinetic descriptors give an acceptable range defined for a lead compound providing for further drug development.

**Figure 12:**
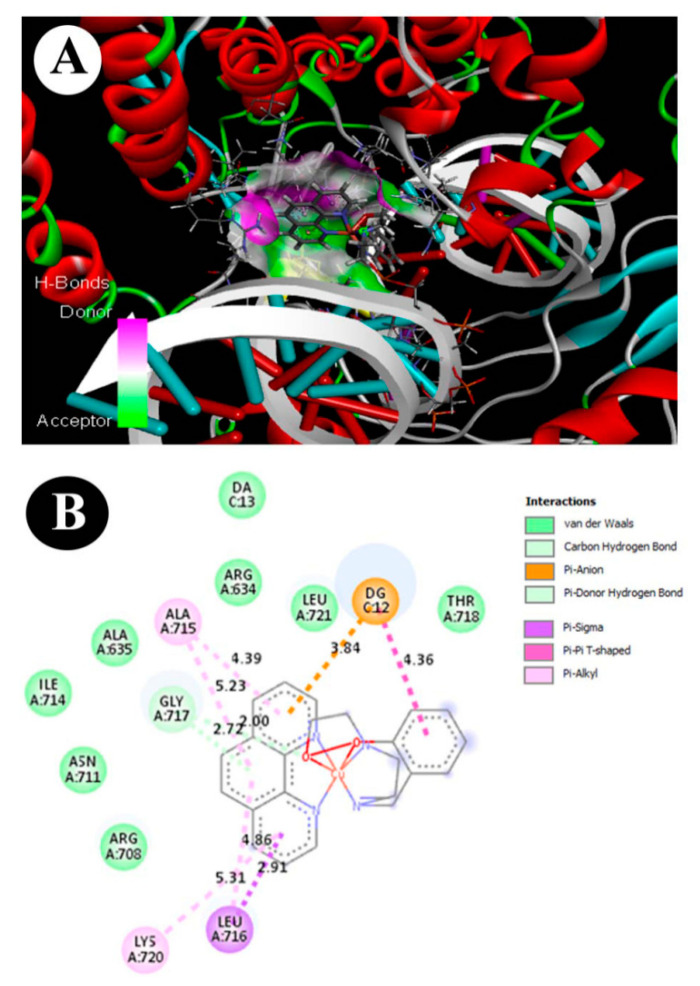
Molecular docking analysis of the copper complex with Top1 in DNA. (**A**) 3D view of copper complex with Top1 in DNA. (**B**) 2D view of copper complex interaction with Top1 in DNA.

The molecular docking of copper complex into active site of Top1 enzyme was performed using Discovery Studio 3.5 and Molegro Virtual Docker software (trial version). The final higher docking score was selected in terms of the ligand–protein–DNA complex with the lowest binding energy (−7.8 Kcal/mol). Bond interactions and distance (Å) of the copper complex with amino acid residues in the active site of Top1 enzyme are presented in Figure 12. It was found that Pi-sigma and Pi–Pi T-shaped interaction between the copper complex and the selective active site of Top1 enzyme occurs at a specific site on Leu A:716 and ALA A:175, respectively. Another important non-covalent bond, i.e., Pi–anion interaction with DNA molecule, was found between tdp and phen of the complex with DG C:12 site of DNA. Overall, the docking results indicate that this critical conformational pose of the ligand–protein–DNA complex might play a role in enzyme inhibition and DNA intercalation.

## 4. Discussion

In general, tumors develop due to deregulation of cell proliferation, which is invariably associated with the inability of cells to undergo PCD [59,60]. Several cytotoxic drugs mediate cell death by modulating key elements of the apoptosis cascade and/or the cellular stress response [61]. The primary objective of the present study has been to find if the copper phenolate complex [Cu(tdp)(phen)]^+^ would be cytotoxic to breast cancer cells of very different origins and which manifest in different forms, the p53-positive & ER-positive MCF-7 and p53-negative & ER-negative MDA-MB-231.

It is an attempt to find if this complex would kill either or both the breast cancer cells, and in the latter case, if the underlying mechanisms would be different between the two since two crucial genes, *p53* and *ER* are involved. The 24 and 48 h IC_50_ values reveal a concentration- and time-dependent cytotoxic effect of the complex. Also, the results suggest that both MDA-MB-231 (p53-negative & ER-negative) and MCF-7 (p53-positive & ER-positive) breast cancer cells are sensitive to the complex by mechanisms selective to each. It is likely that the coordinated structural feature of this complex determines this selectivity. The coordinated phen contributes to partial DNA interaction to manifest the cytotoxic property [26,62,63,64], and the Schiff base ligand would encourage intercalation of phen in the minor groove by engaging in hydrogen-bonding interactions between coordinated -NH- and -OH with the functional groups positioned on the edge of DNA bases [45,65,66].

In our study, the nuclear morphological and cell surface externalization staining results clearly revealed the relationship between the cytotoxic property of a substance with the early- and late events in apoptosis. Annexin V is a marker for early events in apoptosis, so our copper complex induced the early events of apoptosis cascade, where phosphatidylserine is translocated from the inner to outer leaflet of the plasma membrane. On the other hand, changes such as chromatin fragmentation, shrinkage of cells, and formation of apoptotic bodies as revealed in AO-EB staining are a clear indication of late apoptotic events. Thus, a major percentage of the affected cells manifested cytological changes consistent with apoptosis inclusive of both early and late events. This is indicated in the phen present in the complex, which is an effective inducer of apoptosis [26,34,35,36,45].

The shift from preponderance of apoptosis to necrosis, as found in this study, is dependent on the concentration of the complex as well as incubation time; higher doses and longer incubation times are required to switch from apoptosis to necrosis. Importantly, necrosis is as effective a role-player in cancer therapy as apoptosis [67]. The shift from apoptosis to necrosis by several copper complexes is due to an interlinked phenomenon of oxidative stress and disruption of redox homeostasis [45].

Reactive oxygen species, including superoxide anion radical, hydrogen peroxide and hydroxyl radical, are produced within cells under normal aerobic growth conditions, but their production is increased under the influence of external stresses. Several recent reports suggest a role for ROS in drug-induced apoptosis through engagement of downstream proteins involved in the execution of apoptosis [25,68]. Modulation of oxidative stress is an important strategy in anticancer drug development. Intracellular ROS is considered a key mediator of apoptosis and, therefore, production of ROS at toxic levels or at a threshold may provide opportunity for exploitation to kill cancer cells [69]. In this study, the pattern of intracellular ROS level was altered on treatment, differentially between MCF-7 and MDA-MB-231 cells, whereupon apoptosis was induced in both cases. Thus, in the copper(II) phenolate complex-induced cell death, in this study, an imminent role for ROS is indicated. In most cell types, mitochondria produce substantial quantities of ROS when the cell is subjected to stress [70,71].

Concomitant with alteration in ROS levels, there was loss of mitochondrial membrane potential. The ROS production is further derived from the already reported plasmid DNA cleavage effect by [Cu(tdp)(phen)]^+^ in the presence of reducing agent ascorbic acid and also without an added reductant. In fact, the complex [Cu(tdp)(phen)]^+^ exhibited conversion of the supercoiled (SC) form of plasmid DNA to nicked circular (NC) and linear circular (LC) forms through the generation of freely diffusible hydroxyl radical, a reactive oxygen species (ROS), directly responsible for initiation of the cleavage reaction and thus cleaved plasmid DNA in the absence of a reducing agent [51]. Thus, the copper complex brings about evidence to the effect that free oxy-radical species can play an important role in directing the course of cell death. Therefore, it is suggested that the copper complex modulates the intra-cellular redox system and, thereby, provides for accumulation of ROS differentially between MCF-7 and MDA-MB-231 cells, oxidative DNA damage, and cell death.

The activation of endogenous endonucleases, with subsequent cleavage of DNA into oligonucleosomal fragments, is a hallmark of apoptosis [72]. Copper complexes, which are highly oxidized, have been reported to induce DNA strand breaks and base damage in various cancer cells [37,45,51,64,70]. The results of the comet assay in this study indicate DNA damage, thus supporting the inference arrived at from morphological analyses, loss of mitochondrial membrane potential and alteration of intracellular ROS levels, and a majority of affected cells succumbed to death by apoptosis [73].

The results in terms of copper complex-induced nuclear, cell surface and mitochondrial changes are in good correlation with the incidence of apoptosis as revealed in the increase of sub-G1 population. Chemical agents that damage DNA are known to act via modulation of redox system, to produce growth arrest and induce apoptotic cell death [28,29,34,35,51,65,70]. Cell cycle progression is regulated in an orderly manner from G1 to M, through S and G2, by phase-specific regulation of the levels of cyclins, cyclin-dependent kinases (CDKs) and tubulin, and also their inhibitors, which have strong association with apoptosis [74,75]. The data emanating from analysis of cell cycle in this study reveal that there was perturbation in the percentage of cells distributed among the various phases of cell cycle, which is driven by the nature of the complex, the cell type and the duration-dependence.

The different patterns of cell death in this regard in the two different breast cancer cells strongly suggest that the mechanism of action of the complex may be genotype-selective (i.e., p53-positive/negative and ER-positive/negative). Numerous investigations have correlated oxidative stress with different p53-directed cell fates, such as cell cycle arrest, DNA repair and apoptosis [76]. For example, generation of excess ROS in mitochondria, resulting from treatment with chemotherapeutic agents, leads to apoptosis [77,78]. For p53 to effectively regulate the basal levels of ROS, a redox-active regulation loop should exist, which would serve as a channel for cross talk between the basal levels of cellular ROS and p53 and to keep the fluctuations of ROS and p53 within physiological ranges [40,41,77,78].

It is an established fact that p53 is an important regulator of transcription, and more than 300 genes are regulated by this transcription factor. The p53-mediated apoptosis, in response to DNA damage, is predominantly attributable both to transcriptional activation of genes that encode pro-apoptotic proteins (such as Bax) and transcriptional repression of genes that encode anti-apoptotic proteins (such as Bcl-2) [79,80]. The results of this study indicate both ER-positive and ER negative breast cancer cells as susceptible to p53-associated response of our copper complex modulation, fairly different between ER-positive MCF-7 cell and ER-negative MDA-MB-231 cell. This is evidenced by reasonably different shifts in p53 expression and Bax/Bcl-2 protein ratios in the two breast cancer cells. When correlated with the all other parameters (cytotoxicity, morphological changes, annexin V-positivity, DNA damage, change in mitochondrial trans-membrane potential and S-phase & G2/M arrest), both types of cells are sensitive to the complex but via molecular mechanisms essentially cell type-specific. Moreover, estrogen receptor-dependent modulation of gene expression is a potential target for chemotherapeutic agents. Estrogen receptor is directly associated with the control of cell proliferation and cell death induction [81]. However, the ER-p53 sensitivity assessment needs more in-depth gene expression profile analyses that will help us draw a clear conclusion about the mode of action of the copper complex.

More than one pathway, with a redox system as the mediator in both cases, is apparently associated with the course towards apoptosis. Especially, p53 has an integral role in maintaining a mitochondria-mediated redox mechanism as well as DNA repair mechanism. In this context, topoisomerase I is an upstream target to p53 and has direct association with mitochondria-mediated apoptosis and cell cycle inhibition. Several studies have reported that human topoisomerase I (Top1—PDP: 1SC7) is one of the important molecular targets for a number of anticancer drugs. For example, analogues of camptothecins, indolocarbazoles, and indenoisoquinolines inhibit and/or intercalate with Top1-DNA complex [82,83,84]. In our molecular docking studies, a strong binding affinity of copper complex towards Top1 enzyme-DNA complex (−8.5 kcal/mol) was found. Particularly, Pi–sigma and Pi–Pi T-shaped interactions between copper complex and Top1 enzyme occur on Leu A:716 and ALA A:175, respectively. Moreover, Pi–anion interaction with DNA (DG C:12 region) where the residues i.e., Leu A:716, ALA A:175 and DG C:12, might have a key role in the enzyme inhibition and DNA intercalation (Figure 8). High affinity and strong specificity of the copper complex with Top1–DNA complex might play an important role in the inhibition of cancer cell activities such as replication, and alteration in transcription, redox mechanism and DNA repair mechanism with association to the cell death mechanism. Thus, the mechanism of cell death as induced by the copper complex appears to be essentially p53-mediated with at least a redox system in common. From these perspectives, it will be pertinent to work further on the mechanisms of cell death induced by the copper complex adopting ER- and/or p53-based comparative molecular studies. Investigation along this direction is in progress. 

Thus, this study shows that a mixed ligand copper(II) complex, [Cu(tdp)(phen)]^+^) with a diimine co-ligand (phen), is capable of dealing with two genotypically different breast cancer cells in different manners, and is rather selective, culminating in apoptosis and necrosis. This is interesting since ER-negative breast cancer cells have so far found very poor prognostic strategies, and the present finding can go a long way in this direction, provided the complex is subjected to further testing at the molecular level. It is concluded that the copper complex offers potential to be developed as a target-specific anticancer agent for treatment of breast cancers in general and ER-negative ones in particular.

## Figures and Tables

**Table 1:** In vitro MTT cytotoxicity assay for the complex against human breast carcinoma cell lines MCF-7 and MDA-MB-231 [IC_50_ values (Dm), sigmoiditiy (m) and correlation (r)].

Cell Lines	24 h			48 h		
	IC_50_	m	r	IC_50_	m	r
	(µM)			(µM)		
MCF-7	1.6 ± 0.8	2.9	0.8	1.2 ± 0.8	2.8	0.8
MDA-MB-231	1.9 ± 1.2	3.1	0.9	1.0 ± 0.9	2.2	0.9

**Table 2:** Table showing DNA content of cells (MCF-7 and MDA-MB-231) distributed among different phases (sub-G0, G0+G1, S, G2+M) of cell cycle as revealed in FACS analysis. The data shown are means ± SD from three independent replicates.

Complexes	Duration	Sub-G0	SD	G0+G1	SD	S	SD	G2+M	SD
**MDA-MB-231**									
Control	24 h	5.05	±0.6	80.77	±0.29	4.47	±0	7.46	±0.3
[Cu(tdp)phen]+		1.82	±0.2	59.84	±0.46	19.4	±0.1	14.9	±0.2
Control	48 h	5.38	±0.7	83.88	±1.09	3.31	±0.2	5.96	±0.4
[Cu(tdp)phen]+		3.69	±0.3	53.59	±1.02	17.1	±0.2	20.9	±0.4
**MCF-7**									
Control	24 h	2.74	±0.3	77.39	±0.72	6.74	±0.4	9.89	±0.5
[Cu(tdp)phen]+		5.35	±1.8	53.28	±8.88	16.4	±2.5	17.4	±3.7
Control	48 h	2.41	±0.4	54.84	±3.4	22.2	±1.8	16.8	±1.8
[Cu(tdp)phen]+		25.72	±4.5	21.63	±0.31	15.8	±1	28.3	±2.7

**Table 3:** In silico ADMET properties of the copper complex deciphered adopting Discovery Studio prediction software.

Descriptors	Score
ADMET AlogP98 (partition coefficient)	3.588
ADMET Aqueous Solubility	−7.391
ADMET Absorption Level	0
ADMET PSA2D—(Fast polar surface area)	31.909
ADMET EXT PPB	2.89411
ADMET BBB	0.45
ADMET EXT CYP2D6	−2.30385
ADMET EXT Hepatotoxicity	−2.52683
TOPKAT Ames mutagenicity	Non-Mutagen
TOPKAT Ames mutagenicity score	−2.33954
TOPKAT Carcinogenicity in female rat	Non-Carcinogen
TOPKAT Carcinogenicity in male rat	Non-Carcinogen
